# MicroRNA-Mediated Myostatin Silencing in Caprine Fetal Fibroblasts

**DOI:** 10.1371/journal.pone.0107071

**Published:** 2014-09-22

**Authors:** Bushuai Zhong, Yanli Zhang, Yibo Yan, Ziyu Wang, Shijia Ying, Mingrui Huang, Feng Wang

**Affiliations:** 1 Jiangsu Livestock Embryo Engineering Laboratory, Nanjing Agricultural University, Nanjing, PR China; 2 Jiangsu Engineering Technology Research Center of Meat Sheep & Goat Industry, Nanjing Agricultural University, Nanjing, PR China; University of Massachusetts Medical, United States of America

## Abstract

Myostatin functions as a negative regulator of skeletal muscle growth by suppressing proliferation and differentiation of myoblasts. Dysfunction of the myostatin gene, either due to natural mutation or genetic manipulations such as knockout or knockdown, has been reported to increase muscle mass in mammalian species. RNA interference (RNAi) mediated by microRNAs (miRNAs) is a promising method for gene knockdown studies. In the present study, transient and stable silencing of the myostatin gene in caprine fetal fibroblasts (CFF) was evaluated using the two most effective constructs selected from four different miRNA expression constructs screened in 293FT cells. Using these two miRNA constructs, we achieved up to 84% silencing of myostatin mRNA in transiently transfected CFF cells and up to 31% silencing in stably transfected CFF cells. Moreover, off-target effects due to induction of interferon (IFN) response genes, such as interferon beta (*IFN-β*) and 2′-5′-oligoadenylate synthetase 2 (*OAS2*), were markedly fewer in stably transfected CFF cells than in transiently transfected cells. Stable expression of anti-myostatin miRNA with minimal induction of interferon shows great promise for increasing muscle mass in transgenic goats.

## Introduction

Myostatin, a growth and differentiation factor-8 (*GDF-8*), is a member of the transforming growth factor-β (TGF-β) superfamily and is a major regulator of skeletal muscle growth. It is expressed predominantly in skeletal muscles and acts as a negative regulator of skeletal muscle growth by suppressing proliferation and differentiation of myoblasts [1], [2]. Deletion of the myostatin gene in knockout mice leads to increased muscle mass and decreased fat tissue [1], [3], [4]. A phenomenon known as double muscling, caused by natural mutations in the myostatin coding sequence, has been observed in certain cattle breeds such as Belgian Blue, Piedmontese and South Devon [5], [6]. Mutations in the myostatin gene have also been shown to cause double muscling in humans and other species [7]–[9]. These findings suggest that strategies for inhibiting myostatin function may be applied to improve animal growth.

miRNA-mediated gene regulation has recently been shown to be a key regulatory layer controlling gene activity in the cell [10], [11]. miRNAs are short non-coding RNAs that negatively regulate gene expression at the post-transcriptional level [12], [13]. To date, more than 1,000 miRNA genes have been identified in mammalian genomes. These genes are transcribed and processed in the nucleus to generate hairpin loop-structured single-stranded precursor miRNAs (pre-miRNAs) of ∼70 nucleotides in length [14], [15]. Following export from the nucleus into the cytoplasm by exportin-5 [16], [17], the pre-miRNAs are processed by Dicer into a single-stranded mature miRNA of ∼22 nucleotides in length, which is then incorporated into the miRNA-induced silencing complex (miRISC) [18], [19]. The mature miRNA in the RISC complex can bind to target mRNAs with full or partial base complementarity and can downregulate gene expression by translational repression or mRNA cleavage [20].

To date, animal miRNAs have been reported to functionally target endogenous mRNAs only through sites in the 3′-untranslated regions (UTRs), but experiments using artificial sites have shown that target sites for endogenous miRNAs can be identified in open reading frames (ORFs) and 5′-UTRs [21], [22], and genome-wide analyses have suggested that a large amount of targeting, involving thousands of mRNAs, happens in ORFs [23]–[28]. However, endogenous ORF targeting is less frequent and appears less effective than 3′-UTR targeting, but is still far more frequent than 5′-UTR targeting [13], [23]–[25]. The ability to obtain powerful and stable RNAi silencing is critical for many applications, especially for gene knockdown in transgenic animals. However, the off-target effects of long-term stable silencing are still poorly understood because the majority of RNAi studies have focused on short-term transient gene silencing. In addition, numerous experiments have used established cell lines, which may not accurately represent *in vivo* conditions, and possible off-target effects of RNAi in particular [29].

*In vitro* myostatin knockdown in CFF cells using an miRNA-expressing construct would be useful for devising suitable *in vivo* strategies for myostatin knockdown. In turn, this may facilitate production of a transgenic goat with increased muscle mass. At present, few reports are available on the development of miRNA-expressing constructs against myostatin in livestock species. In this study, we examined both transient and stable silencing of the myostatin ORF in CFF cells using miRNA-expressing vectors and analyzed off-target effects due to induction of IFN-responsive genes (*IFN-β*, *OAS2*).

## Materials and Methods

### 1. Ethics Statement

Animal care and use protocols were in strict accordance with the guidelines set by the Animal Research Institute Committee of Nanjing Agricultural University, China. Goats were housed in a temperature-controlled room with appropriate light-dark cycles, fed a regular diet, and maintained under the care of the Laboratory Animal Unit, Nanjing Agricultural University, China. Fetuses were removed from the goats by caesarean section under epidural anesthesia with lidocaine, and then euthanized using carbon dioxide asphyxiation. This study was approved by the Animal Research Institute Committee, Nanjing Agricultural University, China.

### 2. Design of miRNAs and construction of plasmids

**Figure 1:**
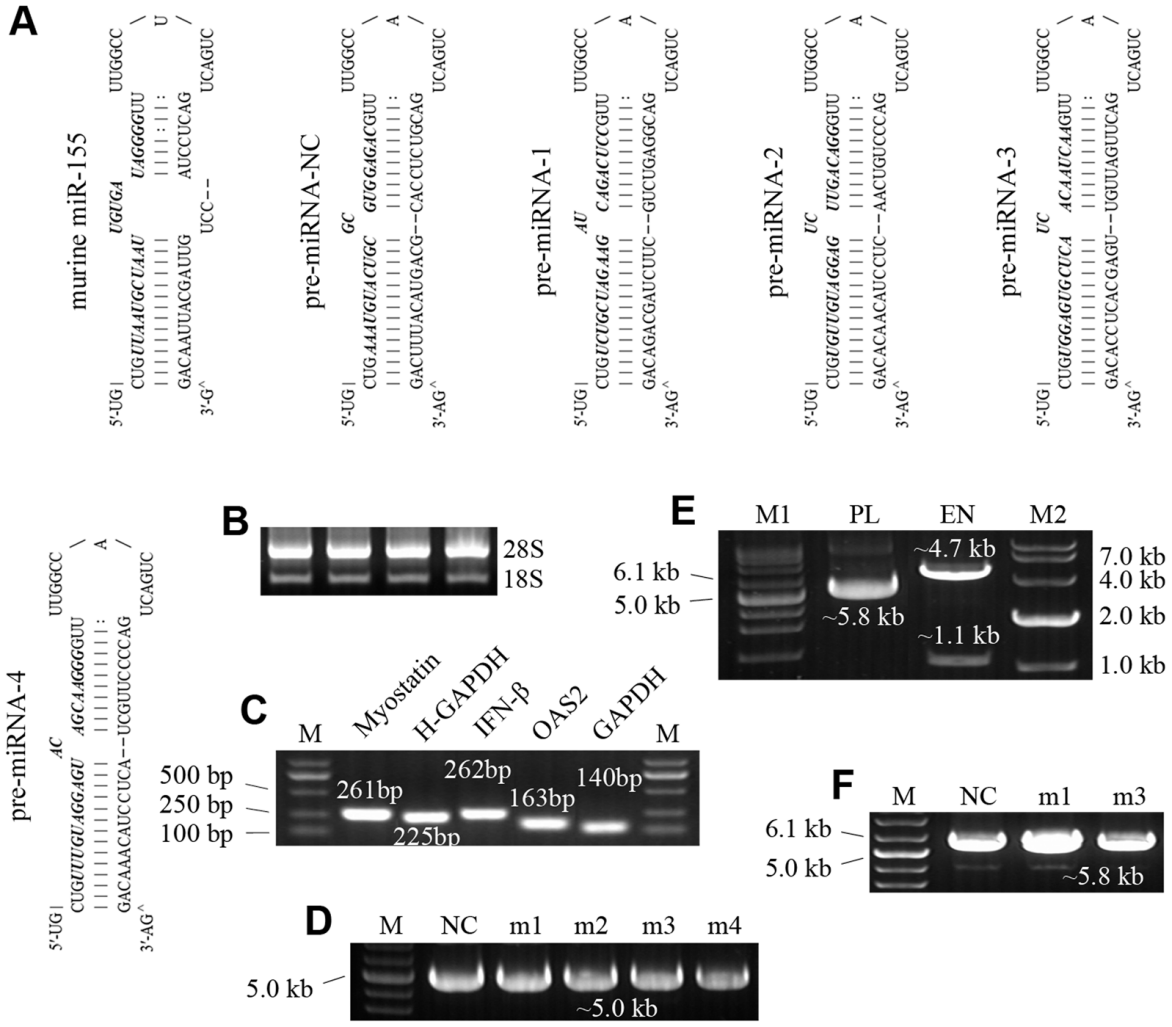
Engineered pre-miRNAs and agarose gel electrophoresis of nucleic acid fragments. (A) Stem-loop structures of engineered pre-miRNAs. Pre-miRNA-1∼4 were designed to target myostatin mRNA. Pre-miRNA-NC was used as the negative control. All pre-miRNAs were based on the murine miR-155 structure. The sequences giving rise to mature miRNA are bold and italicized. Nucleic aid fragments were visualized by the Agarose gel electrophoresis in (B∼F). (B) Total RNA extracted from cells. (C) The products of qPCR for target genes. M: DL2000 DNA Marker. (D) pD-miRNA plasmids. M: Supercoiled DNA Ladder Marker; NC: pD-miRNA-NC; m1∼4: pD-miRNA-1∼4. (E) Restriction enzyme digestion of C1-MSTN. M1: Supercoiled DNA Ladder Marker; PL: C1-MSTN plasmid; EN: the digested products of C1-MSTN by XhoI and BamHI; M2: DL10000 DNA Marker. (F) pDG-miRNA plasmids. M: Supercoiled DNA Ladder Marker; NC: pDG-miRNA-NC; m1: pDG-miRNA-1; m3: pDG-miRNA-3.

**Figure 2:**
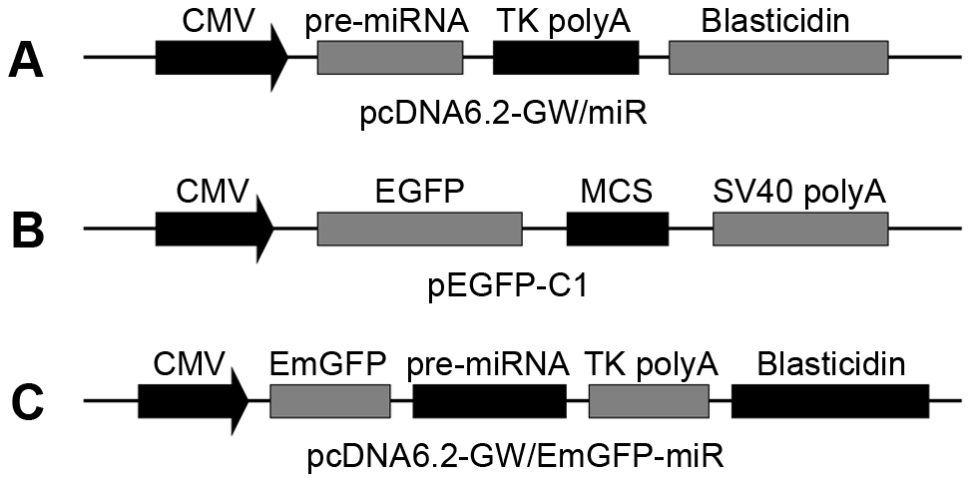
Graphic representation of three kinds of plasmids used in this experiment. (A, C) Plasmids used to express miRNAs. They both contain the blasticidin resistance gene for antibiotic selection of transgenic cells and the cytomegalovirus (CMV) promoter driving expression of miRNAs. Plasmid in (A) is the same as in (C), but with emerald green fluorescent protein (EmGFP) removed. (B) Plasmid used to express the fusion of enhanced green fluorescent protein (EGFP) and target genes cloned into the multiple cloning site (MCS) in eukaryotic cells. TK polyA: thymidine kinase polyadenylation signal; SV40 polyA: simian virus 40 polyadenylation signal.

We designed and synthesized four miRNA mimics of caprine myostatin (gene accession no. AY436347) (National Center for Biotechnology Information; NCBI) along with the negative control that did not knock down mammalian mRNA (Figure 1A). Individual miRNAs were converted to intramolecular stem-loop structures known as pre-miRNAs, which were based on the murine miR-155 sequence. Each pre-miRNA was 64 nucleotides in length, comprising a partial flanking sequence, an miRNA hairpin and a mature miRNA derived from the target gene. Using the BLOCK-iT Pol II miR RNAi expression vector kit (Life Technologies, Carlsbad, CA), we annealed and cloned the oligonucleotides encoding the engineered pre-miRNAs into pcDNA6.2-GW/miR (Figure 2A), to generate pD-miRNA plasmids. All plasmids were sequenced to confirm the insertion of the double-stranded miRNA oligonucleotides. In addition, full-length caprine myostatin was also chemically synthesized and cloned into pEGFP-C1 (Figure 2B), to generate a C1-MSTN plasmid expressing a myostatin and EGFP fusion protein, for monitoring myostatin protein expression.

### 3. Cell culture

Human embryonic kidney (HEK) 293FT cells were purchased from Life Technologies. CFF cells were derived from a 60-day-old fetus of a Huanghuai goat, following the method described by Lu et al. [30]. Briefly, CFF cells were established using the primary explant technique. Both cell lines were cultured in Dulbecco's modified Eagle medium (DMEM) with antibiotics (100 IU/mL penicillin and 100 µg/mL streptomycin) and 10% fetal bovine serum (FBS) in a 37°C humidified atmosphere containing 5% CO_2_.

### 4. Identification of the two most effective miRNAs against myostatin

293FT cells were used to screen the pD-miRNA plasmids. Cells were seeded in six-well plates (Corning Incorporated, Corning, NY) at a density of 60,000 cells/cm^2^ 24 h before transfection. For transfection, individual pD-miRNA plasmids were co-transfected with C1-MSTN into 293FT cells using Lipofectamine 2000 (Life Technologies) at a ratio of 3∶1∶10 (3 µg of pD-miRNA/1 µg of C1-MSTN/10 µL of Lipofectamine). Twenty-four hours after transfection, fluorescence signals were captured under a fluorescence microscope (TE2000-U; Nikon Corporation, Tokyo, JPN). Subsequently, cells were harvested and examined for myostatin expression. To determine myostatin expression, one half of the cells were used for quantitative polymerase chain reaction (qPCR) and the other half were used for western blot analysis.

### 5. Knockdown of myostatin in CFF cells

Based on the above results, the two most effective anti-myostatin miRNA oligos and their negative controls were cloned into pcDNA6.2-GW/EmGFP-miR (Figure 2C) to generate pDG-miRNA plasmids. The miRNA oligos were inserted into the 3′-UTR of the *EmGFP* gene, which facilitates miRNA expression tracking. To study the silencing efficacy of the two plasmids, CFF cells were seeded in 60-mm dishes (Corning Incorporated) at a density of 15,000 cells/cm^2^ 24 h before transfection. Individual plasmids were transfected into CFF cells using Lipofectamine 2000 at a ratio of 1∶2.5 (8 µg DNA/20 µL Lipofectamine). Twenty-four hours after transfection, cells were harvested and examined for myostatin expression, which was determined by qPCR. Furthermore, induction of IFN response by the two anti-myostatin plasmids was monitored by qPCR for two classic IFN-stimulated genes (*IFN-β* and *OAS2*). To further test inhibitory activity, each of the two anti-myostatin plasmids was transfected into CFF cells. Forty-eight hours after transfection, cells were passaged at a dilution of 1∶10 and cultured in the presence of blasticidin to select clones in which the expression of myostatin and the two IFN-stimulated genes was determined by qPCR.

### 6. qPCR analysis

Total RNA (Figure 1B) was extracted from cells using an RNA isolation kit (BioTeke Corporation, BJ, CHN). The concentration of the RNA samples was determined using a NanoDrop 1000 Spectrophotometer (Thermo Fisher Scientific, Wilmington, DE) to ensure equal quantities of RNA. cDNA was synthesized from 1 µg of total RNA using a PrimeScript RT Master Mix (TaKaRa, Dalian, CHN). qPCR analysis was performed on an ABI Prism 7300 system (Life Technologies) using FastStart SYBR Green Master mix (Roche Applied Science, Mannheim, Germany) with cDNA. The sequences of the target genes were derived from either NCBI or the Goat Genome Database (GGD; Chinese Academy of Sciences). Primers specific to the target genes were designed and are shown in Table 1. Amplification specificity was monitored by evaluating the melting curve and examining the PCR products on agarose gels for the absence of non-specific bands (Figure 1C). PCR results were normalized to glyceraldehyde 3-phosphate dehydrogenase (GAPDH) or human GAPDH (H-GAPDH). Gene expression levels were quantitated using the Pfaffi method [31].

**Table 1:** List of primers used for qPCR.

Gene	Primer sequence (5′→3′)	Amplicon	NCBI or GGD accession no.
Myostatin	F-ATCTGAATGAGAACAGCGAGCA	261 bp	AY436347
	R-CGTCTTCCAAGGAGCCGTC		
H-GAPDH	F-TGAAGGTCGGAGTCAACGGAT	225 bp	M33197
	R-CCTGGAAGATGGTGATGGGAT		
IFN-β	F-ACTCCTGGGGCAGTTACATTC	262 bp	NM_001114297
	R-CATTATTTCCTTCTGGATTGGC		
OAS2	F- CGGATAACACCTGCTGGCTAC	163 bp	GOAT_GLEAN_10016578
	R- GGCTGGAGAAAGTCCTTGATG		
GAPDH	F-GCCGCCTGGAGAAACCTAA	140 bp	GOAT_ENSBTAP00000040484
	R-GTGAGTGTCGCTGTTGAAGTCG		

### 7. Western blot analysis

For detecting 30-kDa myostatin-EGFP fusion and 36-kDa GAPDH proteins, 30 µg of protein extract was electrophoresed on 12% SDS polyacrylamide gels, using 1∶5000 mouse monoclonal anti-EGFP antibody (Sunshine Biotechnology, Nanjing, CHN) and 1∶10000 mouse monoclonal anti-GAPDH antibody (LifeSpan BioSciences, Seattle, WA). Goat anti-mouse antibody (Abmart, Shanghai, CHN), linked to horseradish peroxidase (HRP), was diluted 1∶5000 and used as the secondary antibody. Immunoreactive bands were visualized using ImageQuant LAS 4000 (Fujifilm, Tokyo, JPN). Band intensities were estimated by densitometry and normalized to GAPDH. Densitometric analysis of the bands was performed using the Image-Pro Plus (IPP) software version 6.0 (Media Cybernetics, Silver Spring, MD).

### 8. Image analysis

293FT cells transfected with C1-MSTN were photographed using a fluorescence microscope. All settings for image capture were kept consistent and the exposure time for each sample was two milliseconds (ms). Background noise produced by scatter from out-of-focus light and by noise inherent to the imaging system was subtracted from cell fluorescence measurements using the IPP software, which was also used to quantify fluorescence in the images. Averages of fluorescence intensity represent levels of myostatin-EGFP fusion protein.

### 9. Statistical analysis

The mean values and standard deviations (SD) for triplicate samples were calculated using Microsoft Excel (Microsoft, Redmond, WA). Student's *t*-test was performed to identify significant changes. A p-value <0.05 was considered statistically significant.

## Results

### 1. Identification of the two most effective miRNAs against myostatin

**Figure 3:**
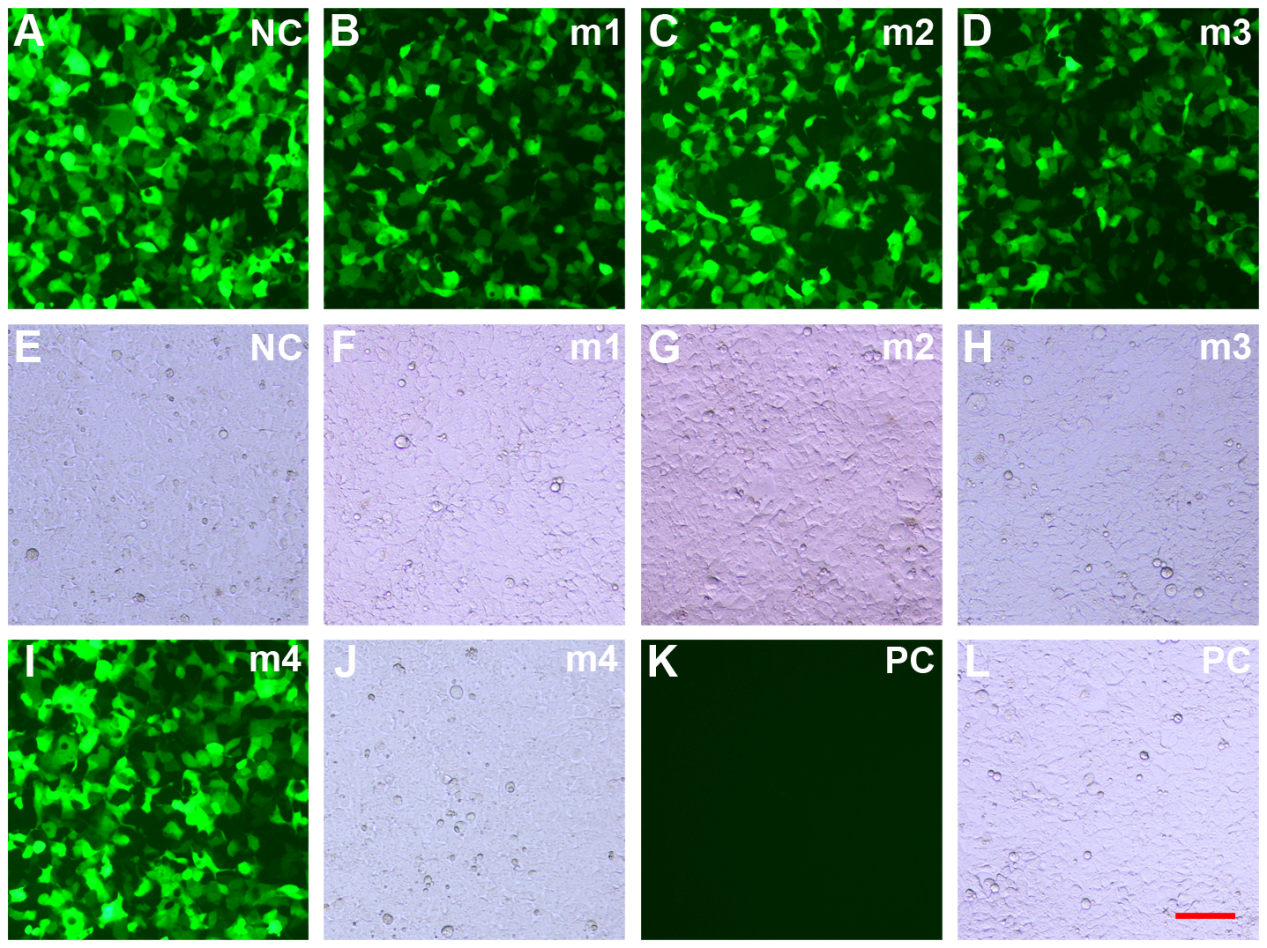
Images of 293FT cells co-transfected with C1-MSTN and each of the pD-miRNA plasmids. Under a fluorescent field, all settings for image capture were kept consistent and the exposure time for each sample was 2 ms. All images were taken at ×100 magnification. (A∼D, I, K) fluorescent light. (E∼H, J, L) visible light. PC: parental 293FT cells control; NC: pD-miRNA-NC; m1∼4: pD-miRNA-1∼4; scale bar: 100 µm.

**Figure 4:**
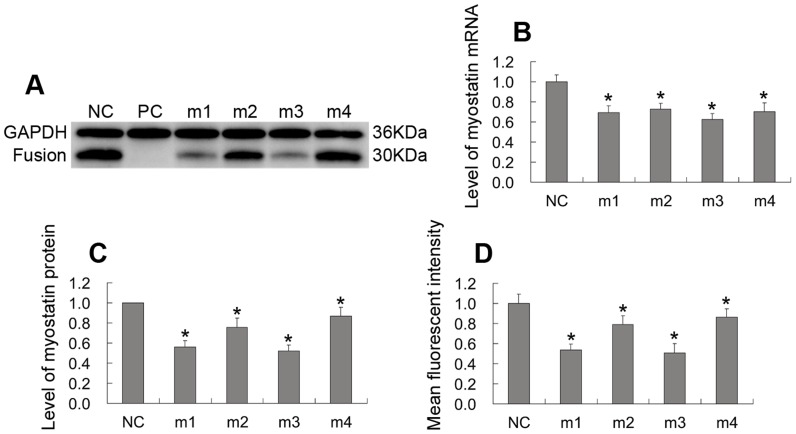
Identification of the optimal anti-myostatin miRNAs in 293FT cells. Each of the four pD-miRNA plasmids was co-transfected with C1-MSTN into 293FT cells. Protein levels of myostatin-GFP fusion were determined by Western blotting (A, C) and mean fluorescent intensity (D). (B) Myostatin mRNA levels were measured by qPCR. GAPDH levels were used as the internal control. NC: pD-miRNA-NC; m1∼4: pD-miRNA-1∼4; PC: parental 293FT cells control. *p<0.05.

Four miRNAs were designed and cloned into the pcDNA6.2-GW/miR plasmid (Figure 2A). To determine the efficacy of these miRNAs for myostatin knockdown, the pD-miRNA plasmids (Figure 1D) were cotransfected with C1-MSTN (Figure 1E, 2B) into 293FT cells. qPCR, western blot analysis and quantification of fluorescence intensity were performed to measure myostatin levels in transfected cells (Figure 3, 4). All miRNAs caused significant downregulation of myostatin expression compared to miRNA-NC (mNC; p<0.05). In addition, miRNA-1 (m1) and miRNA-3 (m3) showed the highest efficacy, reducing myostatin mRNA by up to ∼38% and myostatin protein by ∼48%, respectively. These studies demonstrated that specific and efficient silencing of caprine myostatin can be achieved using miRNA. m1 and m3 were selected as the optimal miRNAs to knock down caprine myostatin in subsequent experiments.

### 2. Silencing of myostatin in CFF cells

**Figure 5:**
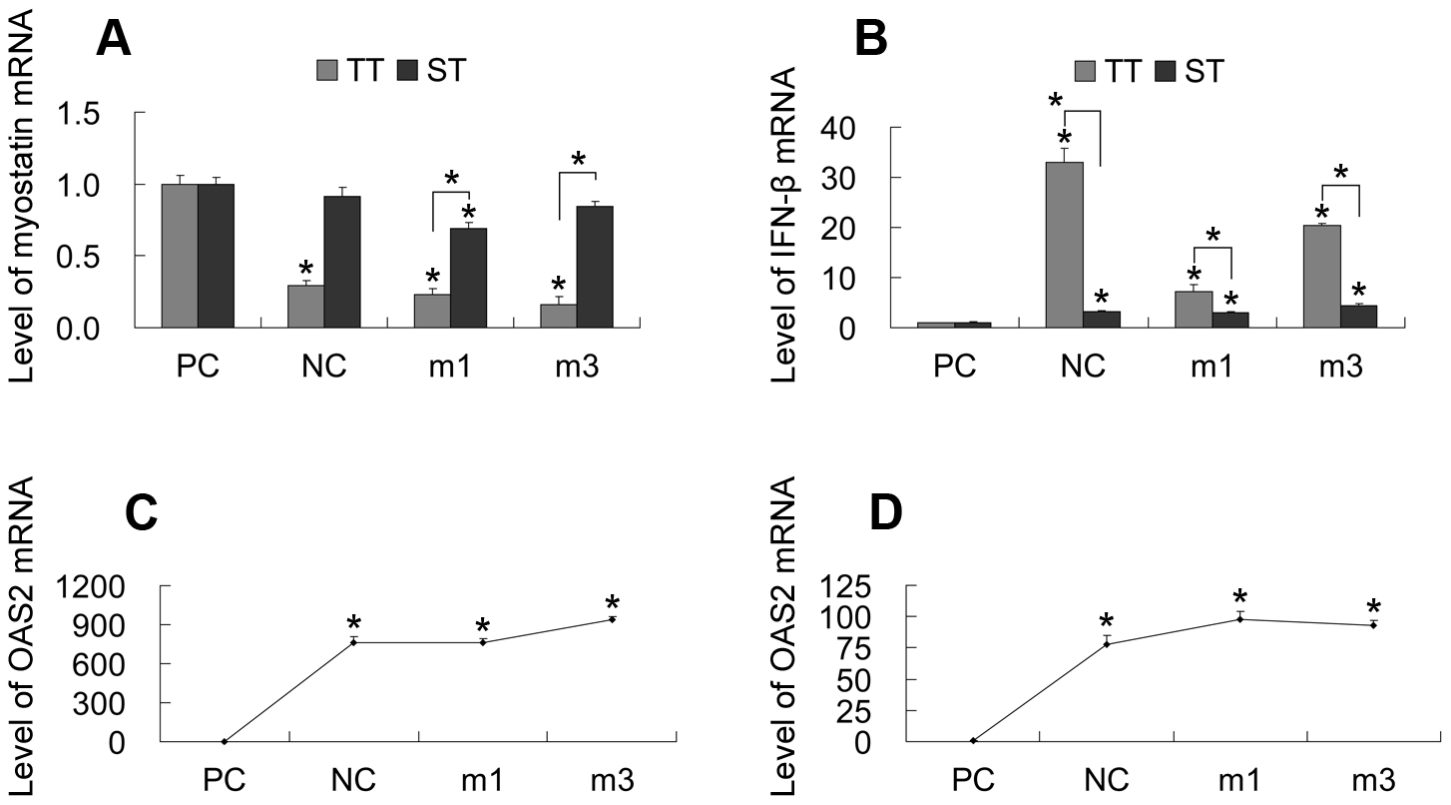
Knockdown of myostatin and induction of the IFN response in CFF cells. Each of the two pDG-miRNA plasmids was transfected into CFF cells transiently (A∼C) and stably (A, B, D). The mRNA levels of myostatin (A) and two IFN stimulated genes (*IFN-β* and *OAS2*) (B∼D) were determined by qPCR, which were normalized to GAPDH levels. PC: parental fetal myoblasts control; NC: pDG-miRNA-NC; m1: pDG-miRNA-1; m3: pDG-miRNA-3; TT: transient transfection; ST: stable transfection. *p<0.05.

**Figure 6:**
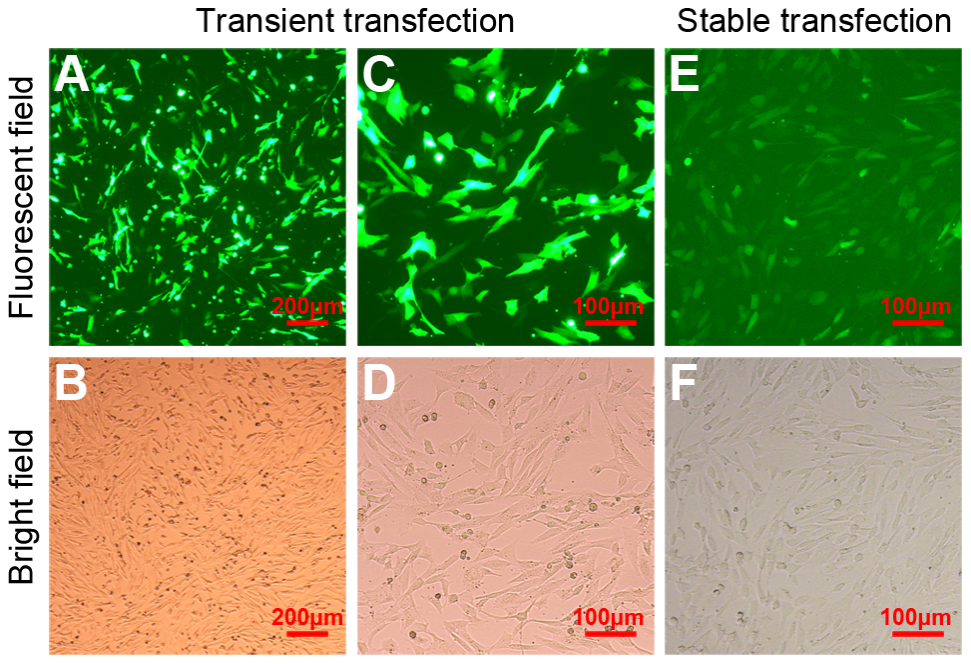
CFF cells in monolayer cultures. (A, C) CFF cells transiently transfected with pDG-miRNA plasmid in a fluorescent field. (B) Cells visualized from (A) in a bright field. (D) Cells visualized from (C) in a bright field. (E) Morphology of CFF cells stably transfected with pDG-miRNA plasmid in a fluorescent field. (F) Cells visualized from (E) in a bright field. Images were taken at ×40 magnification in (A, B) and at ×100 magnification in others. The scale bar was either 200 or 100 µm.

m1 and m3 were cloned into pcDNA6.2-GW/EmGFP-miR plasmids (Figure 2C). The ability of the pDG-miRNA plasmids (Figure 1F) to produce RNAi effects was tested in transiently transfected CFF cells (Figure 5A). Approximately 80% transfection efficiency was achieved, based on the number of cells showing GFP expression under fluorescent light with respect to the total number of cells observed under visible light (Figure 6A–6D). Interestingly, both miRNAs and mNC reduced myostatin mRNA levels by 71–84% (p<0.05) relative to that in non-transfected cells. The ability of RNAi to silence myostatin expression was also tested in stably transfected cells (Figure 5A, 6E, 6F). The results showed that silencing of myostatin by m1 or m3 was less efficient in stably transfected cells than in transiently transfected cells. However, m1 reduced the myostatin mRNA level by 31% relative to that in non-transfected cells (p<0.05); m3 reduced mRNA levels, but the result was not significant (p>0.05). In stably transfected mNC cells, the level of myostatin mRNA was not significantly reduced (p>0.05).

### 3. IFN response in CFF cells transiently and stably transfected with miRNA

Induction of IFN response by anti-myostatin miRNAs was monitored by qPCR for two classic IFN-stimulated genes (*IFN-β* and *OAS2*). Our results showed that all miRNAs, including mNC, induced a 7- to 33-fold induction of *IFN-β* (Figure 5B; p<0.05) and a 759- to 937-fold induction of *OAS2* (Figure 5C; p<0.05) in transiently transfected CFF cells relative to that in the non-transfected control. Similarly, a 2.9- to 4.3-fold induction of *IFN-β* and a 4.1- to 7.2-fold induction of *OAS2* was observed in stably transfected CFF cells (Figure 5B, 5D). Thus, it appears that all miRNAs induced an IFN response in both transiently and stably transfected CFF cells, but that the response was much weaker in stably transfected cells than in transiently transfected cells.

## Discussion

RNAi has become a common tool for controlled downregulation of gene expression in cultured cells, as well as in various model organisms [32]. Furthermore, study of myostatin function has raised the possibility that its inhibition might be useful for increasing muscle mass in agricultural applications. Recently, several researchers have reported efficient RNAi-mediated silencing of myostatin using shRNA vectors in various species [33]–[36]. In particular, fibroblasts have been successfully used to study myostatin expression [37]–[39]. In this study, we demonstrated efficient myostatin gene silencing in CFF cells, although the IFN response, *OAS2* expression in particular, was high against all plasmid constructs.

We detected highly efficient silencing of myostatin, with up to 84% significant reductions in the level of myostatin mRNA in transiently transfected CFF cells and up to 31% reductions in stably transfected CFF cells. Variation in silencing efficiency was observed among the different plasmids, despite their similar ranking and GC contents (five-star ranking; 43–52% GC). Similar variation in silencing efficiency (up to 92%) has been reported in a caprine fibroblast cell line [30], [33]. In recent years, myostatin silencing in fibroblasts has also been demonstrated in other species. Ajai et al. reported a reduction of up to 68% in myostatin transcripts in chicken embryonic fibroblasts by introducing synthetic small interfering RNAs (siRNAs) [32]. Similarly, Stewart et al. observed large variation in silencing efficiency (37–92%) among different shRNA vectors in porcine fibroblasts [38]. Moreover, Hu et al. and Tang et al. reported up to 90% reduction of myostatin expression in sheep fibroblasts using shRNA vectors [40]–[42].

Broadly speaking, RNAi is a process of gene silencing which can be induced by intracellular expression of short hairpin RNAs (shRNAs) or miRNA mimics delivered by vectors. However, shRNAs are not an optimal substrate for RNAi, because shRNAs are not processed by Drosha, an enzyme that creates cleavage sites for further cleavage by Dicer, which is critical for efficient RNAi-induced silencing [12], [43]. Therefore, screening of a large number of candidate shRNA sequences was required to identify the active ones. In addition, shRNA-expressing vectors always use RNA polymerase III (Pol III) promoters, thereby limiting applications such as tissue-specific shRNA expression and increasing the risk of cellular toxicity caused by shRNA overexpression, which can interfere with endogenous miRNA biogenesis [44].

Recently, miRNAs, driven by polymerase II (Pol II) promoters, that become mature miRNAs via a naturally existing endogenous miRNA pathway, have been shown to be more potent inducers of RNAi, with lower toxicity than conventional shRNAs [45]–[47]. Pol II is tightly regulated and can drive tissue-specific miRNA expression. In addition, using miRNA expression constructs driven by Pol II promoters avoids saturation of endogenous miRNA pathways caused by overexpression of exogenous shRNAs [48], [49]. However, these miRNAs have not been applied in the anti-caprine myostatin RNAi strategy. In the present study, four miRNAs targeting myostatin ORF sequences were designed using an online tool. Then, pD-miRNA plasmids expressing these miRNAs driven by Pol II promoters were constructed. In 293FT cells co-transfected with pD-miRNA and C1-MSTN plasmids, we observed that all miRNAs could significantly inhibit myostatin expression via cleavage of their target sequences; m1 and m3 showed the strongest inhibitory effect. Our results indicated that miRNA-mediated RNAi might be a potent method to induce gene silencing.

It has been well established that off-target effects result from gene perturbations caused by unintended interactions between the RNAi molecules and cellular components; this was first reported by Jackson and colleagues in 2003 and was shown to complicate the interpretation of RNAi data [50], [51]. The delivery-associated off-target effects can be divided into three separate categories [52]: first, miRNA-like off-target effects which result from imperfect sequence homology to the unintended targets; second, triggering of an immune response by activation of Toll-like receptors, leading to global degradation of mRNA and general inhibition of protein translation, which result from the RNAi construct or its delivery vehicle; third, overwhelming of the endogenous RNAi machinery (saturation effect) due to the introduction of RNAi molecules, impacting miRNA processing and function. In this study, we observed that mNC, which did not knock down mammalian mRNA, produced a significant reduction (p<0.05) in myostatin mRNA levels in transiently transfected CFF cells, indicating that myostatin inhibition is not the result of miRNA-like off-target effects.

Another potential off-target effect is produced by saturation of the RNAi machinery, especially exportin-5, a nuclear transport receptor. Blockade of the cellular miRNA pathway can result in off-target de-repression of multiple mRNAs and lead to liver toxicity and fatal effects in mice in a dose-dependent manner [44], [53]–[54]. Not surprisingly, exportin-5 overexpression enhances miRNA-mediated RNAi by eliminating the export chokepoint and allowing cells to tolerate higher amounts of miRNA [52], [55]. Because of the competition for exportin-5 and argonaute-2, endogenously produced miRNAs were downregulated, transcripts with seed sites for highly-expressed endogenous miRNAs were significantly upregulated in cells transfected with synthetic miRNAs, and targets containing sites for endogenous miRNAs other than the transfected miRNA exhibited reduced silencing [52]. Thus, the saturation effect should cause upregulation of myostatin expression in CFF cells transiently transfected with mNC; however, we observed the opposite result. This suggests that myostatin inhibition does not result from off-target silencing caused by the saturation effect, and that the saturation effect can be avoided by utilizing miRNA expression constructs driven by Pol II promoters.

Based on the above analyses, the off-target effects that caused mNC transfection to elicit myostatin inhibition in CFF cells are most likely due to the immune response. Several of the early studies to report post-transcriptional gene silencing (PTGS) of myostatin in mammalian fibroblasts demonstrated that RNAi constructs had the potential to elicit off-target gene silencing via induction of the IFN response [32], [38]. These non-specific events are either caused by direct regulation of the target gene by IFN, or by global mRNA downregulation following activation of RNase L [56]. We investigated this phenomenon by measuring the expression of two IFN-induced genes (*IFN-β* and *OAS2*) following transfection with pDG-miRNA plasmids. The results showed that all miRNAs, including mNC, induced statistically significant increases in expression of IFN-stimulated genes in both transiently and stably transfected CFF cells. The IFN response was much weaker in stably transfected cells than in transiently transfected cells, due to the small number of miRNA copies integrated into genomic DNA, resulting in low levels of miRNA expression in stably transfected cells. We also discovered that cells stably transfected with mNC no longer showed a significant reduction in myostatin mRNA levels, and that the silencing efficiency of m1 and m3 against myostatin were significantly reduced in stably transfected CFF cells. These results indicated that although more information is required for standardization of strategies to overcome the immune response and for measurement of the extent to which IFN stimulated genes are upregulated, keeping miRNA doses well below the point at which the IFN response downregulates myostatin mRNA levels can ameliorate these off-target effects.

Because antibody specificity and myostatin sequences differ among species, it is difficult to find an antibody suitable for detecting the silencing of caprine myostatin at the protein level, using western blot analysis. For this reason, some researchers have not detected changes in myostatin protein expression using an RNAi approach [32], [33], [38], [57]. In the present study, the monoclonal anti-EGFP antibody was selected to test myostatin protein expression indirectly in 293FT cells, via the tag-protein method [58], [59]. We demonstrated that m1 and m3 significantly decreased myostatin protein expression by 48%. However, miRNA insertion into the 3′-UTR of the EGFP gene in the C1-MSTN plasmid results in a frameshift mutation within the splice site and thus leads to a truncated 30-kDa myostatin-EGFP protein. Fortunately, this makes transfer of the fusion protein from polyacrylamide gel to polyvinylidene fluoride membranes convenient. In addition, we tried to measure the levels of myostatin-EGFP protein by quantifying fluorescence intensity in captured images. Interestingly, expression of the fusion protein, as measured by image-analysis, was concordant with the results of western blot analysis; the lower mean fluorescence intensity detected in EGFP-positive 293FT cells indicated that myostatin-EGFP expression was decreased and that myostatin expression was inhibited.

In conclusion, CFF cells could be a suitable model to study miRNA-mediated myostatin silencing. To the best of our knowledge, this is the first study to investigate potential long-term knockdown of the myostatin gene in goats. We demonstrated that m1 produces efficient transient and stable silencing of myostatin expression in CFF cells *in vitro*. Off-target effects caused by the IFN response were much weaker in stably transfected CFF cells than in transiently transfected cells. Thus, a cell line stably transfected with m1 could be used to produce donor cells for the creation of goats with increased muscle mass, using somatic cell nuclear transfer technology (SCNT). In addition, further study of the physiological functions of myostatin using RNAi technology, such as identifying its downstream target genes and underlying signaling pathways, both *in vitro* and *in vivo*, would be beneficial.
